# Timing of hepatitis C treatment initiation and retention in office-based opioid treatment with buprenorphine: a retrospective cohort study

**DOI:** 10.1186/s13722-023-00389-8

**Published:** 2023-05-25

**Authors:** Mary L. Geist, Andrea C. Radick, Judith I. Tsui, Kendra L. Blalock, Addy Adwell, Elsabeth Tamru, Nancy C. Connolly, Jocelyn R. James

**Affiliations:** 1 University of Washington School of Medicine, 1959 NE Pacific St, Seattle, WA 98195 USA; 2 Division of General Internal Medicine, Department of Medicine, Harborview Medical Center, University of Washington School of Medicine, 325 Ninth Avenue, P. O. Box 359780, Seattle, WA 98104 USA; 3 Harborview Medical Center, 325 Ninth Avenue, P.O. Box 359780, Seattle, WA 98104 USA

**Keywords:** Hepatitis c treatment, Office-based buprenorphine treatment, Buprenorphine retention

## Abstract

**Background:**

This study examined associations between receipt of hepatitis C (HCV) treatment and retention in office-based opioid treatment (OBOT) care.

**Methods:**

We conducted a retrospective cohort study of HCV-infected patients who initiated OBOT treatment between December 2015 and March 2021 to characterize HCV treatment and assess associations with OBOT retention. HCV treatment was characterized as no treatment, early treatment (< 100 days since OBOT initiation) or late treatment (≥ 100 days). We evaluated associations between HCV treatment and cumulative days in OBOT. A secondary analysis using Cox Proportional Hazards regression was done to determine the rate of discharge over time when comparing those who did versus did not receive HCV treatment as a time-varying covariate. We also analyzed a subset of patients retained at least 100 days in OBOT care and evaluated whether HCV treatment during that period was associated with OBOT retention beyond 100 days.

**Results:**

Of 191 HCV-infected OBOT patients, 30% initiated HCV treatment, of whom 31% received early treatment and 69% received late treatment. Median cumulative duration in OBOT was greater among those who received HCV treatment (any: 398 days, early: 284 days and late: 430 days) when compared to those who did not receive treatment (90 days). Compared to no HCV treatment, there were 83% (95% CI: 33–152%, P < 0.001), 95% (95% CI: 28%-197%, p = 0.002 and 77% (95% CI: 25–153%, p = 0.002) more cumulative days in OBOT for any, early and late HCV treatment, respectively. HCV treatment was associated with a lower relative hazard for discharge/drop-out, although results did not meet statistical significance (aHR = 0.59;95% CI: 0.34–1.00; p = 0.052). Among the subset of 84 patients retained in OBOT at least 100 days, 18 received HCV treatment during that period. Compared to those who did not receive treatment within the first 100 days, those who received treatment had 57% (95% CI: -3%-152%, p = 0.065) more subsequent days in OBOT.

**Conclusions:**

A minority of HCV-infected patients received HCV treatment after initiating OBOT treatment, but those who did had better retention. Further efforts are needed to facilitate rapid HCV treatment and evaluate whether early HCV treatment improves OBOT engagement.

## Introduction

While historically, hepatitis C infection (HCV) in the United States primarily affected the “baby-boomer” birth cohort, the current epidemic of HCV is closely associated with injection drug use related to the opioid epidemic [1]. High transmission and prevalence among people who use drugs (PWUD) make HCV a threat to public health as well as a major contributor to chronic liver disease, multi-system disease, and mortality. Eliminating this public health threat requires identifying and curing HCV among PWUD and is made feasible thanks to significant advances in direct-acting antiviral (DAA) treatment [2–4]. Recent data showing reduced prevalence of HCV viremia after regional scale-up of DAA treatment among people who inject drugs in Scotland provides real-world evidence to support a “treatment as prevention” approach [5].

Oral DAA medications have greatly simplified HCV treatment for the majority of individuals and have cure rates of greater than 95% in clinical trial settings and only slightly lower rates in real-world studies, including among PWUD [6–9]. Among PWUD, receipt of medication for opioid use disorder (MOUD) (e.g. methadone, buprenorphine) is associated with increased HCV treatment [9–11] and decreased risk of reinfection [12]. PWUD have historically faced barriers to HCV treatment, including concerns about adherence and side effects during the interferon-based era [13], as well as absintence requirements that were written into many state Medicaid coverage guidelines for DAAs [14]. More recent guidelines specify PWUD as a priority population for HCV treatment [15]; however, barriers to care remain, including unstable housing or homelessness, superceding social issues, distrust in the medical system and patient concern about treatment side effects [16, 17]. In 2018, Washington State launched a hepatitis C virus elimination campaign that includes a pharmacy policy that removes a requirement for abstinence and other coverage restrictions for Medicaid patients; the state also partnered with AbbVie to provide glecaprevir/pibrentasvir without prior authorization to Medicaid patients using a modified subscription model [18]. Colocalization of HCV and opioid use disorder (OUD) care is an important strategy to improve HCV treatment engagement and outcomes [16]. Whether HCV treatment in turn benefits retention in OUD treatment is largely unknown but of high importance, given that MOUD is life-saving [19]. A recent study found a positive association between HCV treatment and retention at an opioid treatment program (OTP) [20]. Qualitative studies support that HCV cure increases self-efficacy [21, 22], an important component of substance use treatment engagement, and it is possible that this benefit of HCV treatment would contribute to improved retention in substance use care. We aimed to address gaps in knowledge about relationships between HCV treatment and OUD care by evaluating associations between prevalence and timing of HCV treatment and OBOT retention among persons with HCV in a hospital-associated OBOT program.

## Material and methods

### Design overview

We retrospectively examined patients enrolled in an OBOT program between December 2015 and March 2021 who had HCV infection (i.e., a detectable HCV RNA result). We described demographic and clinical characteristics by time of HCV treatment initiation, and we examined associations between HCV treatment, timing of HCV treatment and retention in OBOT care. Our specific research questions were, 1) Do patients who receive HCV treatment have better retention in OBOT, and 2) Among patients who remain in OBOT treatment at least 100 days, do those who receive HCV treatment within that 100-day period have better subsequent retention in OBOT, compared to those whose HCV was not treated within that 100-day period? This second research question was included to address the possibility of reverse causality, i.e., that longer duration of OBOT treatment provides more opportunities for HCV treatment.

### Study setting and participants

The study was conducted within an OBOT program of a safety-net hospital in Seattle, WA (Harborview Medical Center). The OBOT program encompasses four clinical settings: two primary care clinics, a behavioral health program, and a transitional clinic that provides follow-up care and referral for patients without established primary care who have visited the emergency department or been hospitalized. The program provides medication for OUD based on a collaborative care model, utilizing nurse care managers and physicians qualified to prescribe buprenorphine. The study sample consisted of patients who initiated OBOT care between the program’s inception in December 2015 and March 2021 and who had known HCV, diagnosed either prior to or following initiation of OBOT care. We defined initiation of OBOT care as having received at least one buprenorphine prescription from the OBOT program. While we did not intentionally exclude patients on injectable naltrexone, we found that in our OBOT program, all patients with a detectable HCV RNA had received a prescription for buprenorphine. HCV infection was defined as having detectable HCV RNA by laboratory records. Patients with HCV who received a DAA prescription prior to OBOT intake were excluded.

### Data sources

The study utilized data from the electronic health record (EHR) accessed via a clinical and administrative data repository and imported into REDCap. Data elements included demographic information (age at first OBOT intake, sex, race/ethnicity and insurance status), psychiatric diagnoses (depression, bipolar disorder, anxiety, post-traumatic stress disorder, schizophrenia and other psychotic disorders) and major medical diagnoses (human immunodeficiency virus (HIV), diabetes, coronary artery disease, chronic kidney disease and cirrhosis). HCV RNA (result and date) and date of first DAA prescription were based on laboratory and medication data from the EHR. OBOT program data collected for the purposes of reporting to state and federal funders were used to define the sample and characterize OBOT treatment episodes and measures of OBOT retention. The study was reviewed by the University of Washington Institutional Review Board, which determined that it did not qualify as human subjects research.

### Measures

HCV treatment initiation (hereafter, “HCV treatment”) was defined as receipt of a prescription for a DAA medication. Treatment could be provided through on-site providers or through referral to a specialty clinic and could occur at any time following OBOT intake, within the study period. To address our first question (Do patients who receive HCV treatment have better retention in OBOT?), HCV treatment was the primary exposure of interest and was categorized both as a dichotomous variable of any HCV treatment vs no treatment and as a three-level variable according to time between OBOT intake and receipt of first DAA prescription [no HCV treatment after OBOT intake, early HCV treatment (within 100 days of OBOT intake) and late HCV treatment (≥ 100 days after OBOT intake)]. The key outcome of interest was cumulative retention in OBOT, calculated as the sum of the days patients spent in all OBOT treatment episodes (i.e., the days between intake and discharge dates for each treatment episode) within the study period. In this analysis, we did not distinguish between HCV treatment that occurred during OBOT care and HCV treatment that took place in between or following episodes of OBOT care. To address temporal concerns, we performed a secondary survival analysis using a Cox-Proportional Hazards model to evaluate time to first OBOT discharge, with a time-varying covariate for receipt of HCV treatment, classified as treated vs. not treated. Including a covariate for a time-varying exposure was done to control for immortal time bias that could result from misclassifying the follow-up time among participants who did not receive their DAA prescription until some time after the start of their OBOT initiation.

To address our second question (Among patients who remain in OBOT treatment for at least 100 days, do those who receive HCV treatment within that 100-day period have better subsequent retention in OBOT, compared to those whose HCV was not treated within that 100-day period?), our sample was limited to those whose first treatment episode lasted at least 100 days. The exposure of interest was HCV treatment within that 100-day period, and OBOT retention was calculated as cumulative retention beyond the first 100 days of OBOT treatment.

Discharge date was based on OBOT program files and was defined as the date 30 days after the last prescription would be expected to run out for each OBOT episode. Within OBOT treatment episodes, we did not further characterize frequency or patterns of buprenorphine prescriptions or clinic appointments. Within the program, lost to follow up is the most common reason for discharge [23].

### Statistical analysis

Descriptive statistics were calculated for variables of interest. Fisher’s exact tests were used to compare binary and categorical variables by HCV treatment status (no treatment, early treatment and late treatment). Cumulative days in OBOT treatment was analyzed as a count outcome variable in negative binominal regression models to account for over-dispersed outcome data. A variable was included in the models to adjust for the fact that patients contributed different amounts of observation time (i.e., were enrolled in OBOT at different times during the study period). The variable was calculated as the time between OBOT enrollment and the final discharge date, or the date of data extraction if no discharge date occurred (i.e., if the patient remained in OBOT at the conclusion of the study period). A secondary analysis using a Cox Proportional Hazards regression model was done to determine the rate of discharge over time when comparing those who did versus did not receive HCV treatment as a time-varying covariate, and the outcome event defined as time to first discharge occurrence after OBOT initiation. Given the modest sample size and limited outcomes, we pursued a parsimonious model selection strategy. Models were adjusted for HIV status only, as it was the sole covariate that demonstrated statistically significant difference in HCV treatment receipt (p < 0.05; Fisher’s exact tests—results not shown).

Statistical analyses were conducted using Stata statistical software (16.1, StataCorp LLC, College Station, TX). All analyses are reported with 95% confidence intervals and 2-sided tests of the null hypothesis at a significance level of 0.05.

## Results

The study cohort was comprised of 191 OBOT patients with HCV infection with a mean age of 45 ± 12.4 years. Twenty-nine percent of patients were identified as female; for the majority of the study period, the EHR did not provide the option to identify as non-binary or transgender. Seventy-seven percent were white, 15.2% were Black and 7.9% identified as other/multiracial. Mental health disorders were common (Table 1).

**Table 1:** Demographic and clinical characteristics of OBOT patient sample by HCV treatment status (N = 191)

Characteristics, n (%)	Total (N = 191)		No treatment (n = 133)		Any treatment (n = 58)		Early treatment, < 100 days (n = 18)		Late treatment, ≥ 100 days (n = 40)	
Age at first OBOT program start										
20–34	37	19.4%	26	19.5%	11	19.0%	3	16.7%	8	20.0%
35–44	54	28.3%	39	29.3%	15	25.9%	5	27.8%	10	25.0%
45–64	82	42.9%	55	41.4%	27	46.6%	9	50.0%	18	45.0%
65 +	18	9.4%	13	9.8%	5	8.6%	1	5.6%	4	10.0%
Female	56	29.3%	41	30.8%	15	25.9%	5	27.8%	10	25.0%
Race										
White/Caucasian	147	77.0%	103	77.4%	44	75.9%	14	77.8%	30	75.0%
Black/African American	29	15.2%	20	15.0%	9	15.5%	2	11.1%	7	17.5%
Other/multiracial^1^	15	7.9%	10	7.5%	5	8.6%	2	11.1%	3	7.5%
Hispanic/LatinX ethnicity	17	8.9%	14	10.5%	3	5.2%	1	5.6%	2	5.0%
Primary insurance^2^										
Medicaid	120	62.8%	79	59.4%	41	71.9%	13	72.2%	28	70.0%
Medicare	32	16.8%	24	18.1%	8	14.0%	1	5.6%	7	17.5%
Self-pay	3	1.6%	3	2.3%	0	0%	0	0%	0	0%
Other^3^	32	16.8%	24	18.1%	8	14.0%	3	16.7%	5	12.5%
Time of HCV test positive^4^										
Prior to or at OBOT start	119	63.0%	92	69.2%	27	48.2%	14	82.4%	13	33.3%
After OBOT start	70	37.0%	41	30.8%	29	51.8%	3	17.7%	26	66.7%
Medical co-morbidities										
HIV	24	12.6%	11	8.4%	13	22.4%	6	33.3%	7	17.5%
Diabetes	29	15.2%	20	15.0%	9	15.5%	3	16.7%	6	15.0%
Coronary artery disease	24	12.6%	14	10.5%	10	17.2%	3	16.7%	7	17.5%
Chronic kidney disease	31	16.2%	20	15.0%	11	19.0%	4	22.2%	7	17.5%
Cirrhosis	25	13.1%	14	10.5%	11	19.0%	4	22.2%	7	17.5%
Psychiatric co-morbidities										
Depression	58	30.4%	37	27.8%	21	36.2%	10	55.6%	11	27.5%
Anxiety/post-traumatic stress disorder	108	56.5%	78	58.6%	30	51.7%	9	50.0%	21	52.5%
Schizophrenia/psychotic disorder	17	8.9%	16	12.0%	1	1.7%	0	0.0%	1	2.5%
Bipolar disorder	38	19.9%	27	20.3%	11	19.0%	4	22.2%	7	17.5%
Median duration in OBOT in days (IQR)	146	(58–382)	90	(46–216)	398	(157–854)	284	(157–796)	430	(148–860)

^1^“Other” includes American Indian or Alaskan Native, Asian, or Native Hawaiian or other Pacific Islander

^2^Percentages do not total to 100% due to missing data

^3^Other includes “other,” “commercial,” and “worker’s compensation”

^4^Two patients who received treatment did not have a test date

One hundred thirty-three (69.6%) people with HCV did not have evidence of receiving a DAA prescription during the study period. Fifty-eight patients (30.4%) did receive HCV treatment with DAAs, namely ledipasvir/sofosbuvir, velpatasvir/sofosbuvir and glecaprevir/pibrentasvir. Of these, 18/58 (31.0%) patients received early treatment and 40/58 (69.0%) received late treatment. There were no significant differences in demographic characteristics or psychiatric conditions according to HCV treatment status (none, early, late). HIV was more common in the early HCV treatment group (6/18, 33.3%) than among those who received no HCV treatment (11/131, 8.4%) or late HCV treatment (7/40, 17.5%) (p = 0.009). The majority (63.0%) of patients had a positive HCV test prior to or at their first OBOT start; having a positive test prior to OBOT start was more common in the early treatment compared to the late treatment group (82.4% v. 33.3%; p < 0.001). Among the total cohort of OBOT patients with HCV infection, 44/191 (23.0%) had more than one OBOT treatment episode, 84/191 (44.0%) were retained in OBOT care for at least 100 days, and the median cumulative duration of OBOT care was 146 days (interquartile range or IQR, 58–382). Median cumulative duration of OBOT was longer among those who received HCV treatment (any, early and late) when compared to those who did not receive treatment (Table 1).

For the entire cohort of 191 patients, negative binomial regression models of cumulative days in OBOT according to HCV treatment status used no HCV treatment as the reference group. Compared to no HCV treatment, there were 83% (95% CI: 33–152%, P < 0.001), 95% (95% CI: 28–197%, p = 0.002) and 77% (95% CI: 25–153%, p = 0.002) more cumulative days in OBOT for any, early and late HCV treatment, respectively. The Cox-Proportional Hazards model using a time-varying covariate for HCV treatment demonstrated that HCV treatment was associated with a lower relative hazards for discharge/drop-out, although results did not meet statistical significance (aHR = 0.59;95% CI: 0.34–1.00; p = 0.052).

Among the 84 patients with HCV who were retained at least 100 days in OBOT, a negative binomial regression model was used to examine cumulative OBOT retention beyond 100 days according to whether patients received HCV treatment within that 100-day period, using no HCV treatment within 100 days as the reference group. Compared to those who did not receive treatment within the first 100 days, those who received treatment had 57% (95% CI: − 3–152%, p = 0.065) more subsequent days in OBOT.

## Discussion

In this retrospective cohort study of patients with HCV enrolled in an OBOT program at a single academic medical center, patients who received HCV treatment following OBOT enrollment had greater cumulative retention in OBOT over the follow-up period compared to those who had not received HCV treatment. OBOT retention was highest for HCV treatment that occurred “early,” i.e., within 100 days of OBOT enrollment, but only a small minority of patients received treatment within that time frame. In a survival analysis to better account for temporality of HCV treatment and OBOT care, HCV treatment was associated with a lower relative hazards for discharge/drop-out, although the results did not reach statistical significance. Similarly, among patients with HCV who were retained at least 100 days in OBOT, those who received HCV treatment within that 100-day period (n = 18) had greater subsequent OBOT retention than did those who were treated later or not treated, although this finding was also not statistically significant.

A recent case–control study by Severe et al. evaluated patients with HCV who were engaged in care at an opioid treatment program (OTP) and found that compared to those who did not receive HCV treatment, those whose HCV was treated at the OTP were 2.22 times more likely to be retained in OTP care for the duration of the study period [20]. Although our study evaluated patients in an OBOT program and had multiple methodological differences compared to the study by Severe et al. we found a similar degree of association between receipt of HCV treatment and retention. That, in our study, the group of patients who received HCV treatment early had the highest cumulative days in OBOT, might suggest that prompt HCV treatment is more strongly associated with retention in OBOT. This is important because OBOT programs provide access to evidence-based, life-saving treatment with buprenorphine for people with OUD [24]; expanding OUD treatment is a key strategy to address overdose deaths in the U.S., which now number more than 100,000 annually [25].

The 191 patients with detectable HCV RNA in our sample comprised 22.4% of the total population of 854 patients who initiated OBOT treatment during the study period. The true number of patients with hepatitis C viremia was likely higher, as we found that only 70.8% of our patients were screened. Though we did not have rigorous processes in place to ensure universal HCV screening, we have since implemented such protocols. For comparison, a prior study by Carey et al [26] found that 32% of patients in an OBOT program had detectable HCV RNA.

We found that 30.4% of patients with HCV received HCV treatment after enrolling in OBOT. While this figure compares favorably to a study of patients enrolled in an OBOT program between 2003 and 2013 (i.e., prior to and early in the DAA era), when only 2.21% of viremic patients were found to have received HCV treatment [26], it is only slightly higher than that found among a non-treatment seeking population of people who inject drugs and have HCV in Seattle, WA, based on a 2018 survey. [27] We also found that although 63.0% of patients were diagnosed with HCV before or at OBOT start, only a small minority received early HCV treatment. We believe that this suggests missed opportunities to provide prompt treatment, which is important to reduce viral transmission and is now explicitly recommended by guidelines [15]. Emergency department or inpatient stays for PWUD may provide opportunities to facilitate early HCV treatment through HCV testing, initial HCV treatment evaluation (e.g., HBV antigen screening and tests to assess fibrosis/cirrhosis) and linkages to care, although evidence to date suggests ongoing gaps between screening and linkage to care, especially for PWUD [28, 29], and further study is needed.

It is notable that of 17 patients with psychotic disorders in our sample, only one (2.5%) received HCV treatment. This important HCV care gap may be due to limited HCV treatment availability at our mental health based OBOT clinic, cognitive and/or communication barriers to medical care among people with psychotic disorders [30], fragmentation of the healthcare system [31] or other factors. However, specific data on access and barriers to DAA treatment for people with psychotic disorders is lacking, and further study is needed. Importantly, real-world evidence among people with HIV and HCV co-infection suggests that DAA therapy is effective among people with psychotic disorders [32].

On the other hand, patients with HIV were over-represented in the any and early HCV treatment groups in this study. In our hospital system, people with HIV receive care in a dedicated clinic with infectious disease trained providers with expertise managing HCV and with more wraparound services than other settings, which could lead to improved OBOT retention. However, when adjusted for HIV status, the relationship between HCV treatment and OBOT retention persisted.

There were limitations to this study. First, the study was observational and cannot establish a causal relationship between HCV treatment and OBOT retention. It is possible that this association is explained by residual confounding factors, e.g., personal or clinical characteristics that contribute to engagement or perceived engagement in both addiction and HCV care. Additionally, the design of our study did not allow us to account for temporal relationships between HCV treatment and OBOT retention among our full cohort, nor did it allow us to distinguish patients whose HCV was treated during OBOT care from those whose HCV was treated following or in between episodes of OBOT care. Our analysis of HCV treatment vs. no treatment in the first 100 days and OBOT retention beyond 100 days among the 84 patients who were retained in OBOT for at least that duration addresses these temporal concerns, including the concern of reverse causality. The analysis found a consistent positive association that did not reach statistical significance. Our analyses were limited by a modest sample size with a limited number of patients who received HCV treatment and a small number of patients who were treated early. In our view, relationships between addiction and HCV treatment are most likely bi-directional, and our study leaves open the possibility that early HCV treatment promotes retention in OBOT care.

Additional limitations of our study include reliance on the EHR (treatment for HCV that occurred outside of the healthcare system could have been missed) and use of data from a single site and period of time. Changes in professional society guidelines, DAA availability and costs, insurance coverage and prescribing restrictions occurred during the study period, and this data may not accurately reflect current care. Results may also not be generalizable to other settings with differing coverage and prescribing policies—e.g., many states’ Medicaid policies continue to restrict access to HCV treatment, including for PWUD [33], whereas treatment of HCV among high-risk populations and addressing stigma are explicit aims of Washington State’s initiative to eliminate HCV [34].

## Conclusions

In this retrospective study of OBOT patients with HCV infection, patients who received HCV treatment had greater retention in OBOT. More research is needed to explore how HCV and OUD care can be leveraged to optimize treatment of both conditions. Controlled and/or larger observational studies that can account for temporal ordering would be helpful to clarify whether there is a causal relationship between HCV treatment and OBOT retention and to better define the role of early HCV treatment.

## Data Availability

The datasets used and/or analyzed during the current study are available from the corresponding author on reasonable request.
